# Use of high and very high dose radiotherapy after radical prostatectomy for prostate cancer in the United States

**DOI:** 10.1038/s41391-018-0066-5

**Published:** 2018-08-07

**Authors:** P. Alexidis, W. Guo, J. E. Bekelman, N. Vapiwala, P. E. Gabriel, J. P. Christodouleas

**Affiliations:** 1 Interbalkan Center, Thessaloniki, Greece; 2 Department of Radiation Oncology, University of Pennsylvania, Philadelphia, PA USA; 3 Department of Biostatistics, University of Pennsylvania, Philadelphia, PA USA

## Abstract

**Background:**

The cost-benefit tradeoff of radiation dose-intensification for prostate cancer in the post-prostatectomy setting is difficult to predict and is ideally studied in randomized trials. The purpose of this study was to assess the use of dose-escalated post-operative radiation (PORT) for prostate cancer in the United States, during a period in which there were no published level 1 studies on dose-escalation.

**Methods:**

We performed analyses on pT2-3, N0, M0 prostate cancer patients who received PORT after an R0-1 resection within the National Cancer Data Base (NCDB), 2003–2012. We classified patients according to the use of high dose (>66.60 cGy) and very high dose (>70.20 cGy) radiation. We used regression analysis to assess the association of year of treatment with use of high and very high dose PORT. To demonstrate the potential of a registry-based network like the NCDB to prospectively monitor changes in radiation dosing patterns, we determined the year in which a significant change in dose could have been first detected had dose been actively monitored.

**Results:**

Between 2003 and 2012, the use of high dose PORT increased from 29.9% CI (26.7–33.1) to 63.5% CI (60.6–66.5) and very high dose PORT from 4.5% CI (3.1–5.9) to 10.8% CI (8.9–12.6) (adjusted *p* < 0.01, for both trends). Patients diagnosed at community centers were less likely to be treated with high dose PORT compared to those at academic or comprehensive centers (*p* < 0.01 for both comparisons). Had the NCDB network been prospectively monitoring PORT dose, significant increases in dose would have been detected as early as 2004 and after every year of the study period.

**Conclusions:**

The use of both high dose and very high dose PORT increased two-fold from 2003 to 2012 in the absence of randomized studies. This change in practice may be exposing patients to excess toxicity without cancer control benefits. Monitoring dosing patterns using cancer registries is feasible.

## Introduction

Incremental technical advances in linear accelerators and in image guidance have steadily improved the delivery of radiotherapy to a patient’s target volume while sparing the adjacent normal tissues. However, in post-operative radiation (PORT) for prostate cancer, dose escalation cannot be achieved without increasing dose to normal tissues since most cells within the clinical target volume are in fact part of normal/uninvolved nearby organs. As such, dose escalation in this setting invariably increases risks of toxicities. The clinical benefits of dose escalation, however, are not clear. While some retrospective studies suggest dose-escalated PORT may improve biochemical control [1–6], there have been no randomized studies to support it. There does appear to be a clinical benefit from dose-intensification for intact prostate cancer, but even here the results of randomized trials have been challenging to predict [7, 8]. Moreover, randomized trials of dose-intensification in other setting, such as lung and brain cancers, have failed to validate presumed benefits [9–11].

We hypothesized that because the theoretical rationale for dose-intensification is compelling, these treatments are slowly adopted even in the absence of level 1 evidence. To test this hypothesis, that radiation dose creep occurs, we assessed the use of high dose (>66.60 cGy) and very high dose (>70.20 cGy) in PORT for prostate cancer in the U.S. between 2003 and 2012. In addition, we hypothesized that the U.S.’s cancer registry network as represented by the National Cancer Database (NCDB) could be used to prospectively monitor radiation dosing patterns of care and provide early signals of dose creep. To assess the potential of such a registry-based monitoring system, we determined the year in which a significant change in dose could have been first detected had dose been actively monitored.

## Materials/subjects and methods

### Data sources

We analyzed data extracted from the NCDB, which captures information from ~70% of all newly diagnosed cancers in the United States, for the years 2003–2012. We excluded patients diagnosed prior to 2003 because those cases would have been coded prior to the publication of the Facility Oncology Registry Data Standards manual. NCDB analyses were exempted by the institutional review board (IRB).

### Patients

**Fig. 1:**
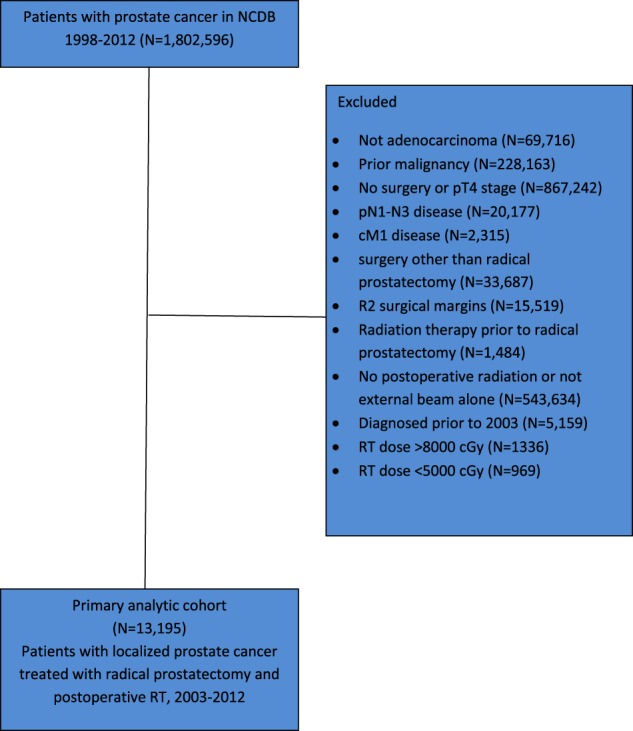
The primary analytic cohort and reasons for exclusion

The details of the cohort selection criteria are shown in Fig. 1. Briefly, we included men that had radical prostatectomy and PORT for prostate adenocarcinoma. To minimize the chances of inadvertent inclusion of patients with gross residual disease after surgery (i.e. an R2 resection), we restricted the cohort to patients with pT2-T3 stage and no pathologically involved pelvic nodes. We excluded patients who had prior chemotherapy or RT which may affect a physician’s selection of dose. In addition, we excluded patients with documented total doses of <5000 cGy and >8000 cGy since these are substantially outside of commonly used doses and may represent patients who discontinued treatment early or have erroneously coded values. Of note, the NCDB does not include information on a patient’s post-operative pre-PORT prostate specific antigen (PSA) level, so we could not distinguish between adjuvant and salvage therapy.

### Primary outcomes and control variables

We defined high dose PORT as >6660 cGy since 6660 cGy was the highest dose used in published randomized trials assessing adjuvant radiation [12–14]. In addition, we defined very high dose PORT as >7020 cGy, the highest acceptable dose identified in our review of guidelines and on-going clinical trials [15–19]. We used year of diagnosis as a surrogate of year of treatment. We identified potential patient and disease covariates that might affect a physician’s view of the tolerability of radiation and characteristics of the diagnosing facility that may be related to patterns of care (Table 1). Facility characteristics were defined as previously described [20, 21]. We used logistic and linear regression to assess the extent that these covariates confound the relationship of year of treatment and use of high and very high dose radiation. We calculated confidence intervals around the estimated annual percentages of patients treated with high and very high dose PORT using the Clopper–Pearson method. We used logistic regression to assess the association of year of treatment (continuous variable) with high and very high dose PORT (categorical variable) adjusting for covariates. We included potential confounders in our final adjusted model if they were associated with year of treatment or use of high or very high dose radiation on univariate analysis with a *p* < 0.10 or if there was a strong clinical rationale for confounding.

**Table 1:** The association of patient and hospital characteristics and year of treatment

	Analytic cohort	*p*-value for the association with the year of treatment^a^
Age, mean (range) in years	64 (25–89)	<0.01
Pathologic stage, No (%)		<0.01
T2	3614 (27.4)	
T3	9581 (72.6)	
Gleason score, No (%)		<0.01
≤6	1283 (9.7)	
7	6354 (48.1)	
8–10	4435 (33.6)	
Missing	1123 (8.5)	
Pre surgical PSA, No (%)		<0.01
<10 ng/ml	7901 (59.9)	
10–20 ng/ml	2235 (16.9)	
>20 ng/ml	1512 (11.5)	
Missing	1547 (11.7)	
Surgical margins, No (%)		0.44
Positive	8817 (66.8)	
Negative	4378 (33.2)	
Hormone therapy, No (%)		<0.01
Yes	3878 (29.4)	
No	8968 (68.0)	
Unknown	349 (2.6)	
Race, No (%)		0.01
White	10819 (82.0)	
Black	1766 (13.4)	
Other	610 (4.6)	
Hispanic origin, No (%)		<0.01
Spanish	579 (4.4)	
Non-Spanish	11399 (86.4)	
Unknown	1217 (9.2)	
Facility location, No (%)		0.35
East	5017 (38.0)	
West	2579 (19.5)	
Central north	4051 (30.8)	
Central south	1548 (11.7)	
Charlson-Deyo score, No (%)		<0.01
0	11158 (84.6)	
1	1805 (13.7)	
2+	232 (1.8)	
Facility type, No (%)		<0.01
Community and other	1456 (11.0)	
Comprehensive	7537 (57.2)	
Academic	4202 (31.8)	
Hospital setting, No (%)		0.77
Metro	10778 (81.7)	
Non-metro	2036 (15.4)	
Missing	381 (2.9)	
Median income, No (%)		0.66
<38,000	1863 (14.1)	
38,000–47,999	2821 (21.4)	
48,000–62,999	3671 (27.8)	
>63,000	4664 (35.3)	
Missing	176 (1.3)	
Insurance status, No (%)		<0.01
No insurance	240 (1.8)	
Private	8432 (63.9)	
Medicaid	338 (2.6)	
Medicare	3755 (28.5)	
Other	238 (1.8)	
Unknown	192 (1.5)	

*PSA* prostate specific antigen

^a^Chi square test for categorical and *t* test for continuous variables

We sought to demonstrate the sensitivity of a hypothetical radiation dose monitoring program within the NCDB network. To determine the year in which a significant change in dose could have been detected had dose been prospectively monitored within the NCDB, we used linear regression to compare the average annual radiation dose (continuous variable) per year with the average of the prior 3 years with adjustments for potential confounders. For 2003 and 2004, where three years of prior data were not available, we forecasted based on the prior year and two years, respectively.

Because multiple hypotheses were being tested, we a priori set statistical significance for the primary analyses at 0.01 using Bonferroni’s method.

## Results

**Fig. 2:**
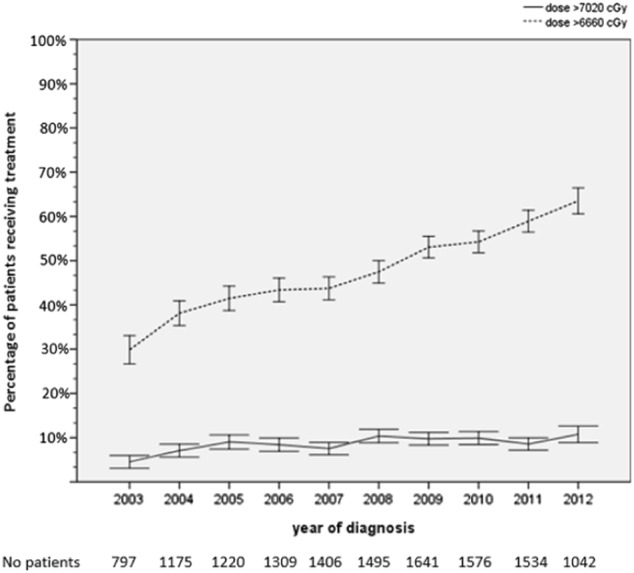
Use of high and very high dose post-operative radiation after radical prostatectomy for prostate cancer, 2003–2012. Bars represent 95% confidence intervals

Our analytic cohort included 13,195 men (Fig. 1). Table 1 shows the distribution of patient and facility characteristics. In the NCDB cohort, the use of high dose PORT increased monotonically from 29.9% CI (26.7–33.1) in 2003 to 63.5% CI (60.6–66.5) in 2012 (Fig. 2). The use of very high dose PORT increased non-monotonically from 4.5% CI (3.1–5.9) in 2003 to 10.8% CI (8.9–12.6) in 2012 (Fig. 2). When dose was analyzed as a continuous variable, the mean value in 2003 was 6377 cGy CI (6331–6422) and 6829 CI (6810–6846) in 2012. In our analyses of potential confounders of the use of high dose PORT, age, pathologic T stage, Gleason score, presurgical PSA, use of hormone therapy, race, Hispanic origin, Charlson-Deyo score, facility type, facility setting and insurance status were included in our final model as covariates because they were potentially associated (*p* < 0.10) with either year of treatment or use of high dose PORT (Tables 1 and 2). A similar set of covariates was included in the model of very high dose radiation, except facility setting did not meet criteria for inclusion and median income did (Tables 1 and 3). Interestingly, surgical margin status was neither associated with year of treatment nor use of high or very high dose radiation (Tables 1–3). Nonetheless, we included it as a covariate in both multivariate regressions because of the clinical rationale for dose escalating in the setting of positive margins, a common oncologic principle in other disease settings.

**Table 2:** The univariate and multivariate association of year of treatment and use of high dose (>6660 cGy) post-operative radiation in the treatment of prostate cancer

	<6660 cGy	>6660 cGy	Univariate		Multivariate	
			OR (CI)	*p*-value^a^	OR (CI)	*p*-value^a^
Year of treatment			1.14 (1.12–1.15)	<0.01	1.14 (1.12–1.16)	<0.01
Age, mean (range)	60.6(37–89)	60.6 (25–87)	1.00 (1.00–1.01)	0.79	0.99 (0.98–1.00)	0.06
Pathologic T stage, No (%)			1.01 (0.90–1.09)	0.70	0.90 (0.80–1.00)	0.07
T2	1877 (27.5)	1737 (27.2)				
T3	4941 (72.5)	4640 (72.8)				
Gleason score, No (%)			0.92 (0.90–0.96)	0.01	0.98(0.93–1.00)	0.50
≤6	666 (9.8)	617 (9.7)				
7	3263 (47.9)	3091 (48.5)				
8–10	2168 (31.8)	2267 (35.5)				
Missing	721 (10.6)	402 (6.3)				
Pre-surgical PSA, No (%)			0.94 (0.91–0.97)	<0.01	1.03 (1.00–1.06)	0.13
<10 ng/ml	4064 (59.6)	3837 (60.2)				
10–20 ng/ml	1092 (16)	1143 (17.9)				
>20 ng/ml	746 (10.9)	766 (12)				
Missing	916 (13.4)	631 (9.9)				
Surgical margins, No (%)			0.97 (0.90–1.00)	0.37	0.95 (0.80–1.00)	0.20
Positive	4580 (67.2)	4237 (66.4)				
Negative	2238 (32.8)	2140 (33.6)				
Hormone therapy, No (%)			1.09 (1.02–1.20)	0.01	1.10 (1.07–1.20)	<0.01
Yes	1944 (28.5)	1934 (30.3)				
No	4703 (69.0)	4265 (66.9)				
Unknown	171 (2.5)	178 (2.8)				
Race, No (%)			0.97 (0.90–1.00)	0.29	0.91(0.86–0.92)	0.01
White	5572 (81.7)	5247 (82.3)				
Black	918 (13.5)	848 (13.3)				
Other	328 (4.8)	282 (4.4)				
Hispanic origin, No (%)			0.95 (0.89–1.00)	0.05	0.98 (0.93–1.00)	
Spanish	328 (4.8)	251 (3.9)				
Non-Spanish	5843 (85.7)	5556 (87.1)				
Unknown	647 (9.5)	570 (8.9)				
Charlson-Deyo score, No (%)			1.00 (0.93–1.09)	0.79	0.97 (0.89–1.05)	0.53
0	5777 (84.7)	5381 (84.4)				
1	916 (13.4)	889 (13.9)				
2+	125 (1.8)	107 (1.7)				
Facility type, No (%)			1.15 (1.09–1.21)	<0.01	1.11 (1.05–1.18)	<0.01
Community+other	873 (12.8)	583 (9.1)				
Comprehensive	3832 (56.2)	3705 (58.1)				
Academic	2113 (31.0)	2089 (32.8)				
Hospital setting			0.88 (0.80–0.97)	0.01	0.90 (0.82–1.00)	0.05
Metro	5502 (80.7)	5276 (82.7)				
Non-metro	1100 (16.1)	936 (14.7)				
Missing	216 (3.2)	165 (2.6)				
Facility location			0.99 (0.96–1.02)	0.86		
East	2563 (37.6)	2454 (38.6)				
West	1377 (20.2)	1202 (18.8)				
Central north	2185 (32.0)	1866 (29.3)				
Central south	693 (10.2)	855 (13.4)				
Insurance status			1.01 (0.98–1.03)	0.39	1.02 (0.99–1.05)	0.12
No insurance	129 (1.9)	111 (1.7)				
Private	4381 (64.3)	4051 (63.5)				
Medicaid	185 (2.7)	153 (2.4)				
Medicare	1911 (28.0)	1844 (28.9)				
Other	109 (1.6)	129 (2.0)				
Unknown	103 (1.5)	89 (1.4)				
Median income			1.00 (0.97–1.04)	0.65		.
<38,000	992 (14.8)	871 (13.8)				
38,000–47,999	1418 (21.1)	1403 (22.3)				
48,000–62,999	1909 (28.4)	1762 (28.0)				
>63,000	2403 (35.7)	2261 (35.9)				

*PSA* prostate specific antigen, *OR* odds ratio, *CI* confidence interval

^a^Chi square test for categorical and *t* test for continuous variables

**Table 3:** The univariate and multivariate association of year of treatment and use of very high dose (>7020 cGy) post-operative radiation in the treatment of prostate cancer

	<7020 cGy	>7020 cGy	Univariate		Multivariate	
			OR (CI)	*p*-value*	OR (CI)	*p*-value^a^
Year of treatment			1.05 (1.03–1.07)	<0.01	1.05 (1.03–1.08)	<0.01
Age, mean (range)	60.6 (25–89)	60.6 (38–85)	1.01 (1.00–1.02)	0.06	1.01 (1.00–1.02)	0.05
Pathologic stage, No (%)			0.86 (0.76–0.99)	0.03	0.82 (0.72–0.95)	<0.01
T2	3266 (27.1)	348 (30.0)				
T3	8770 (72.9)	811 (70.0)				
Gleason score, No (%)			0.93 (0.86–1.00)	<0.01	0.94 (0.86–1.00)	0.19
≤6	1165 (9.7)	118 (10.2)				
7	5793 (48.1)	561 (48.4)				
8–10	4021 (33.4)	414 (35.7)				
Missing	1057 (8.8)	66 (5.7)				
Pre-surgical PSA, No (%)			0.96 (0.90–1.00)	0.01	1.00 (0.94–1.06)	0.93
<10 ng/ml	7190 (59.7)	711 (61.3)				
10–20 ng/ml	2053 (17.1)	182 (15.7)				
>20 ng/ml	1358 (11.3)	154 (13.3)				
Missing	1435 (11.9)	112 (9.7)				
Surgical margins, No (%)			0.95 (0.83–1.08)	0.45	0.94 (0.82–1.07)	0.41
Positive	8054 (66.9)	763 (65.8)				
Negative	3982 (33.1)	396 (34.2)				
Hormone therapy, No (%)			1.13 (1.01–1.26)	0.03	1.19 (1.06–1.33)	<0.01
Yes	3498 (29.1)	380 (32.8)				
No	8219 (68.3)	749 (64.6)				
Unknown	319 (2.7)	30 (2.6)				
Race, No (%)			0.96 (0.85–1.08)	0.42	0.97 (0.86–1.10)	0.50
White	9857 (81.9)	962 (83.0)				
Black	1625 (13.5)	141 (12.2)				
Other	554 (4.6)	56 (4.8)				
Hispanic origin, No (%)			1.06 (0.96–1.16)	0.49	1.07 (0.97–1.18)	0.11
Spanish	525 (4.4)	54 (4.7)				
Non-Spanish	10411 (86.5)	988 (85.2)				
Unknown	1100 (9.1)	117 (10.1)				
Charlson–Deyo score, No (%)			1.03 (0.89–1.18)	0.01	1.00 (0.87–1.15)	0.92
0	10192 (84.7)	966 (83.3)				
1	1624 (13.5)	181 (15.6)				
2+	220 (1.8)	12 (1.0)				
Facility type, No (%)			0.90 (0.82–0.99)	<0.01	0.90 (0.82–1.00)	0.05
Community and other	1342 (11.1)	114 (9.8)				
Comprehensive	6807 (56.6)	730 (63.0)				
Academic	3887 (32.3)	315 (27.2)				
Hospital setting, No (%)			0.96(0.81–1.14)	0.69	.	.
Metro	9828 (81.6)	950 (82.0)				
Non-metro	1862 (15.5)	174 (15.0)				
Missing	346 (2.9)	35 (3.0)				
Facility location, No (%)			1.03 (0.97–1.01)	0.25		
East	4587 (38.1)	430 (37.1)				
West	2339 (19.4)	240 (20.7)				
Central north	3702 (30.8)	349 (30.1)				
Central south	1408 (11.7)	140 (12.1)				
Insurance status, No (%)			0.99(0.95–1.04)	0.46	0.97 (0.92–1.03)	0.34
No insurance	220 (1.8)	20 (1.7)				
Private	7699 (64)	733 (63.2)				
Medicaid	310 (2.6)	28 (2.4)				
Medicare	3405 (28.3)	350 (30.2)				
Other	223 (1.9)	15 (1.3)				
Unknown	179 (1.5)	13 (1.1)				
Median income, No (%)			0.94 (0.88–0.99)	0.05	0.93 (0.88–0.99)	0.03
<38,000	1690 (14.2)	173 (15.1)				
38,000–47,999	2542 (21.4)	279 (24.4)				
48,000–62,999	3364 (28.3)	307 (26.9)				
>63,000	4281 (36)	383 (33.5)				

*PSA* prostate specific antigen

^a^Chi square test for categorical and t test for continuous variables

Year of treatment was significantly associated with use of high (*p* < 0.01) and very high (*p* < 0.01) dose PORT, even after adjusting for confounders (Tables 2 and 3). Because pre-surgical PSAs and Gleason Scores were not available in 2003, we repeated the analysis excluding that year and found similar results.

We analyzed the effect of facility type on patterns of care. Patients diagnosed at community (and other) facilities were significantly less likely to be treated with high dose PORT compared to those diagnosed at academic or comprehensive community facilities (*p* < 0.01 for both comparisons, Table 2). In contrast, patients diagnosed at academic facilities were significantly less likely to be treated with very high dose PORT compared to those diagnosed at comprehensive community facilities (*p* < 0.01, Table 3). Diagnosis at a comprehensive community facility was associated with the highest overall use of high and very high dose PORT.

**Fig. 3:**
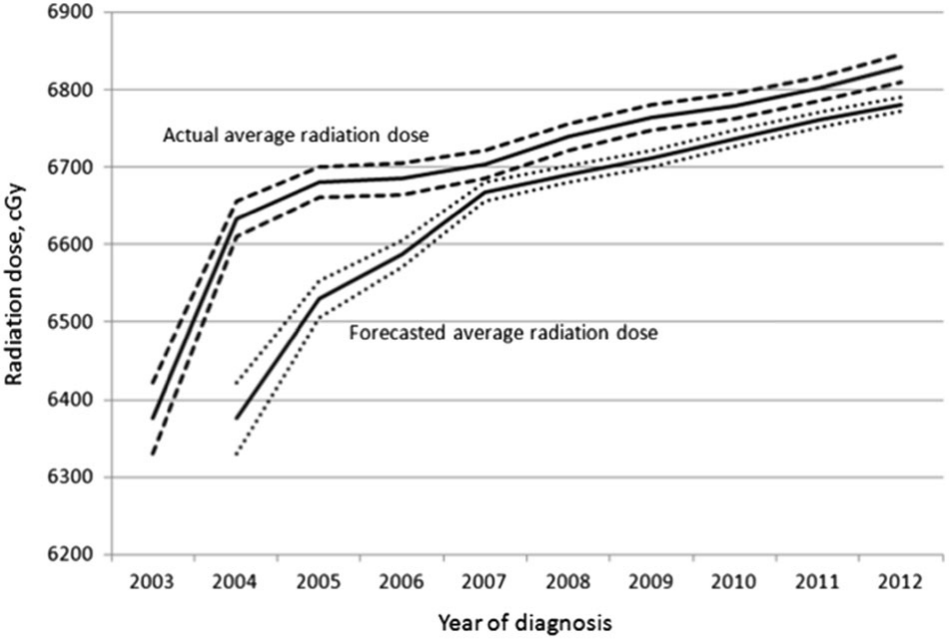
Actual average radiation dose over time versus the forecasted averaged radiation dose (long dashed line). The forecasted averaged radiation dose for a given year was calculated based on the previous three years, except for 2004 and 2005 where it was based on the previous one and two years, respectively. The dashed lines represent 95% confidence intervals

In a proof of principle analysis, we determined the year in which our hypothetical dose monitoring system would have detected a significant change in this cohort. Using an adjusted 3-year rolling average, we found that a significant increase in average dose would have been first detected in 2004 and that a significant change would have been detected after every year in the study period had the NCDB been prospectively monitoring dose in this cohort (Fig. 3).

## Discussion

We found that the use of high and very high dose PORT for prostate cancer increased in the U.S. between 2003 and 2012. By 2012, ~2 out of 3 post-prostatectomy patients were treated with doses >6660 cGy and 1 out of 9 were treated with doses >7020 cGy. This observation is consistent with our hypothesis that radiation dose creep occurred in the absence of level I evidence.

There is retrospective evidence in support of dose escalated PORT > 6660 cGy, particularly in the salvage setting. Ohri et al. [22] performed a meta-analysis with radiobiologic modeling using data from 25 retrospective series of salvage PORT with median PORT doses that ranged from 60 to 72 Gy. They found that salvage PORT dose was an independent predictor of 5-year bPFS and estimated that this endpoint improves 2.5% per additional 100 cGy. In a prior modeling study, King and Kapp [1] found that the tumor control probability dose-response curves for intact prostate cancer and salvage PORT were similar. The dose to achieve 50% biochemical tumor control was 6590 cGy versus 6680 cGy for intact prostate radiation and salvage PORT respectively. Of note, the estimated dose to achieve 50% biochemical tumor control was approximately 600 cGy less for patients treated in the adjuvant PORT setting. The retrospective data to support doses > 7020 cGy is substantially more limited. Cozzarini et al. [3] retrospectively compared outcomes of salvage PORT patients treated with < 7020 cGy vs. ≥ 7020 cGy. Five-year biochemical progression free survival favored the high dose group (83 vs. 71%). In contrast, Goenka et al. [23], found that a salvage PORT dose >70 Gy was not associated with improved biochemical control in patients without evidence of gross residual disease after surgery. To our knowledge, there is presently no randomized evidence supporting dose escalation. SAKK 09/10 is a randomized trial comparing 6400 cGy versus 7000 cGy of salvage PORT [24]. It completed accrual in 2014, but cancer control outcomes have not yet been reported.

The generally favorable results of the retrospective studies of dose escalated PORT could also be explained by confounding. As observed in our NCDB analysis, increasing year of treatment is likely also associated with PORT dose in these retrospective series because dose creep occurs slowly over time. As such, the outcomes of these studies may suffer from stage migration. Improvements in pre- and post-operative imaging over time may better detect patients with gross residual disease after surgery, involved pelvic nodes or distant metastases, which, without any change in treatment, would result in an apparent improvement in cancer control outcomes for PORT patients classified as R0-1, N0, M0. In addition, the retrospective literature may suffer from ascertainment bias since there is a greater opportunity to document a failure event in patients with longer follow-up. Patients treated at higher doses may also have been favorably selected since physicians may be more likely to use novel approaches in the healthiest candidates. Another major weakness of the existing retrospective literature is the use of a biochemical control endpoint. In a disease that is diagnosed in older men and that typically has a long natural history, biochemical control may not be valid surrogate of more clinically meaningful endpoints, which to our knowledge, have not been extensively studied.

There is also evidence that escalating PORT doses are associated with worse toxicity outcomes. The rationale for such a relationship is strong since PORT clinical target volume includes at-risk normal tissue. In 2015, Ghadjar et al. reported the initial acute toxicity results of the SAKK 09/10 randomized trial. Patients in the high dose salvage PORT arm reported a more pronounced and clinically relevant worsening in acute urinary symptoms, though differences in clinician- reported acute genitourinary (GU) and gastrointestinal (GI) toxicities were not significantly different. In a meta-analysis of 25 retrospective series of salvage PORT by Ohri et al., estimates of late grade 3 + GU and GI toxicity ranged from 1–11% to 0–9%, respectively. PORT dose was an independent predictor of both late grade 3 + GU and GI toxicity. Estimated risk increased at a rate of 0.8 and 1.2% per 100 cGy for GU and GI late grade 3 + toxicity. It should be noted that of the 13 studies included in this meta-analyses of toxicities, nine had four or fewer years of toxicity follow up. The long-term toxicity experience of PORT patients is largely unknown.

The data dictionary (FORDS Manual) for NCDB field that codes radiation modality (i.e., intensity modulated radiation therapy (IMRT), 3D conformal, etc.) has inconsistencies that make it unreliable. In particular, it allows registrars to select from options that are not mutually exclusive (e.g., IMRT versus Photons (6-10MV)). As such, we did not include modality as a variable in our analysis. Nonetheless, we suspect that the increasing availability of IMRT in the study period was an important driver of the increasing use of high and very high dose PORT. A recent analysis that used Medicare claims data to ascertain IMRT utilization rates in the United States found that the use of IMRT in the post-operative treatment of prostate cancer increased from zero to 82% between the years 2000 and 2009 [25]. This increase in IMRT utilization parallels the PORT dose trends observed in our study.

In short, the existing retrospective literature may be misleading and the results of randomized comparisons like SAKK 09/10 may be surprising. Something akin to this may have occurred in the radical treatment of locally advanced non-small cell lung cancer. In that disease, the retrospective evidence overwhelmingly favored dose escalation and dose creep was observed in the U.S between 2003 and 2010 [26]. Yet, when the first randomized comparison was reported in 2011, it demonstrated a substantial worsening of toxicity and overall survival in the dose-escalation arm [27].

Another goal of this study was to assess the potential of a prospective national cancer registry-based monitoring system to detect dosing patterns of care and provide early signals of dose creep. In a proof-of-principal analysis, we found that an increasing mean dose of radiation for this cohort could have been detected within the Commission on Cancer’s (CoC) NCDB network as early as 2004 and that a significant change would have been detected after every year in the study period. As such, a national infrastructure for monitoring radiation dosing patterns of care in this cohort and others already exists. Many centers in the U.S. now participate in the CoC’s credentialing program and are already extracting data on radiation dose. The CoC, SEER and other programs that aggregate cancer registry data could feasibly monitor dose to identify cohorts for which the patterns of care are changing in the absence of high level evidence.

This study has important limitations. The NCDB only captures cases where PORT was used as part of the first course of therapy [28]. Patients treated with PORT due to late post-operative biochemical recurrences may have different patterns of care. In addition, we cannot distinguish between patients receiving adjuvant versus salvage therapy as part of the first course of therapy. If these patients are treated differently and their relative proportions are changing over time, changes in dosing may be obscured or magnified. We are also unable to identify patients treated on a clinical trial. It may be that the dose trends we identified are in part reflective of increasing research interests in dose-escalation. Finally, though the NCDB captures 70% of U.S. cancer cases, patients from small and rural facilities may be under represented.
